# Analysis of Serum Metabolites to Diagnose Bicuspid Aortic Valve

**DOI:** 10.1038/srep37023

**Published:** 2016-11-15

**Authors:** Wenshuo Wang, Aikebaier Maimaiti, Yun Zhao, Lingfei Zhang, Hongyue Tao, Hui Nian, Limin Xia, Biao Kong, Chunsheng Wang, Mofang Liu, Lai Wei

**Affiliations:** 1 Department of Cardiac Surgery, Zhongshan Hospital, Fudan University. 200032, Shanghai, China; 2 State Key Laboratory of Molecular Biology, Shanghai Key Laboratory of Molecular Andrology, Institute of Biochemistry and Cell Biology, Shanghai Institutes for Biological Sciences, Chinese Academy of Sciences, 200031, Shanghai, China; 3 Department of Radiology, Huashan Hospital, Fudan University. 200040, Shanghai, China; 4 Department of Chemistry, Laboratory of Advanced Materials, Fudan University, 200433, Shanghai, China

## Abstract

Bicuspid aortic valve (BAV) is the most common congenital heart disease. The current study aims to construct a diagnostic model based on metabolic profiling as a non-invasive tool for BAV screening. Blood serum samples were prepared from an estimation group and a validation group, each consisting of 30 BAV patients and 20 healthy individuals, and analyzed by liquid chromatography-mass spectrometry (LC-MS). In total, 2213 metabolites were detected and 41 were considered different. A model for predicting BAV in the estimation group was constructed using the concentration levels of monoglyceride (MG) (18:2) and glycerophospho-N-oleoyl ethanolamine (GNOE). A novel model named Zhongshan (ZS) was developed to amplify the association between BAV and the two metabolites. The area under curve (AUC) of ZS for BAV prediction was 0.900 (0.782–0.967) and was superior to all single-metabolite models when applied to the estimation group. Using optimized cutoff (−0.1634), ZS model had a sensitivity score of 76.7%, specificity score of 90.0%, positive predictive value of 80% and negative predictive value of 85.0% for the validation group. These results support the use of serum-based metabolomics profiling method as a complementary tool for BAV screening in large populations.

Bicuspid aortic valve (BAV) is a congenital defect in which two of the three leaflets present in the aortic valve are fused together leading to a bicuspid configuration and impaired ability of the heart to maintain a unidirectional blood flow from the ventricle to the aorta. It is the most common type of cardiac valvular deformity and can be found in 0.5% to 2% of the total population^1^.

Despite its prevalence and association with a variety of other cardiovascular abnormities such as aortopathy^2^, early detection of BAV is difficult and conclusive diagnosis usually has to be achieved by an echocardiogram that directly visualizes the heart chambers and valve configuration. While echocardiograms scan are generally non-invasive and radiation-free, they can only be performed individually and as a result are not compatible with automation. Moreover, accurate interpretation of the imaging results often depends heavily on the skill levels of medical practitioners. Therefore, the development of a fast and simple screening method that can be conducted in an unbiased, high-throughput format is highly desired for prompt diagnosis of BAV.

Genetic testing has been successfully employed in the screening of a variety of diseases such as mitral valve prolapse and aortic valve stenosis^3^,^4^. Garg *et al*. in 2005 provided the first experimental evidence indicating the involvement of *NOTCH1*, a gene that encodes a single-pass transmembrane receptor, in the pathogenesis of various developmental aortic valve defects including BAV^5^. Subsequently, Laforest and colleagues demonstrated that Gata5-knockout mice developed partially penetrant BAV and that the same cardiac dysfunction could also result from simple deletion of the same gene from endothelial cells^6^. There has also been research that suggests the implication of several other genes, such as transforming growth factor–receptor types I and II (TGFBR1 and TGFBR2)^7^. Despite these advances, the exact molecular mechanism for BAV pathogenesis remains stubbornly elusive, largely due to the involvement of an intricate regulatory network that governs cardiac development not only at the transcriptional level, but also at the translational and posttranslational levels. As a consequence, this created significant technological hurdles for the development of gene-based diagnostic tests for BAV screening.

Metabolite profiling has in recent years emerged as a powerful analytic tool for the identification of disease-related biomarkers and pathways that can be used to develop new diagnostic and treatment methods^8^. Herein we report the metabolic profiling of the blood serum samples obtained from BAV patients and healthy individuals. The results of the study suggested that a predominant majority of the identified metabolites showing statistically significant concentration differences between the patient group and the control group were mainly clustered in several key pathways, including those associated with fatty acid metabolism, adenosine triphosphate (ATP) degradation, purine metabolism and endocannabinoid biosynthesis. Of particular significance is that we constructed a regression model based on the concentrations of several key metabolites and demonstrated that it could be used for accurate BAV diagnosis. Our serum-based diagnostic method can serve as a complement to echocardiogram for BAV screening, particularly in large populations.

## Methods and Materials

### Patient selection and specimen acquisition

**Figure 1:**
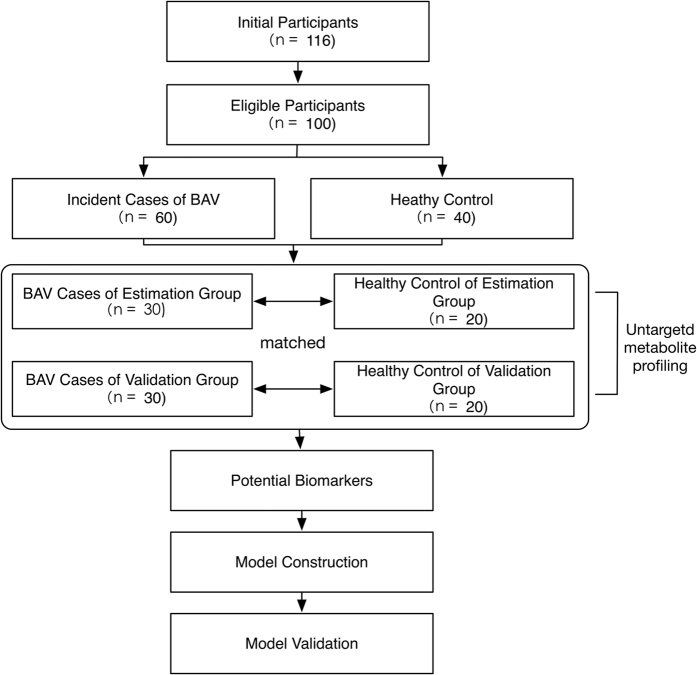
Flow of participants.

Our participants comprised 76 patients diagnosed with BAV by echocardiography and confirmed in subsequent surgery of aortic valve replacement and 40 healthy individuals who underwent physical examination at Fudan University affiliated Zhongshan Hospital from Feb 2014 and May 2015. The guideline used for determining valve dysfunction is “AHA/ACC Guideline for the Management of Patients With Valvular Heart Disease”^9^. All experimental procedures, including collecting serum specimens from patients during the pre-operation period and healthy individuals at health check-ups, were carried out according to the guidelines approved by the Ethics Committee of Zhongshan Hospital and informed patient consent (Reference Number: 20131105002). Informed consent was obtained from all subjects. The exclusion criteria included medicine usage, diabetes, lung diseases, impaired ejection fraction, heart failure, vascular infection, and hypotension. The final pool of eligible participants were divided randomly and equally into an estimation group and a validation group. Each whole-blood sample was centrifuged at 1000 rpm for 15 min to obtain the serum. All serum specimens were stored in liquid nitrogen until metabolomics analysis (Fig. 1).

### Sample treatment

The frozen serum samples were thawed under room temperature. A 100 μL of the serum sample was mixed with 300 μL of methanol (HPLC grade, Merck, Darmstadt, Germany), thoroughly vortexed for 30 s, and subsequently centrifuged at 12000 rpm and 4 °C for 15 min to pellet the protein contents. Once the centrifugation was complete, 200 μL of the supernatant was transferred to a clean sample vial for subsequent analysis. A quality control sample was prepared by pooling equal volumes of blood sera from patients and controls.

### Separation, detection and identification of blood serum metabolites on liquid chromatography-mass spectrometry (LC-MS)

All LC-MS analyses were performed on a 6530 Accurate-Mass Q-TOF LC/MS System with Agilent 1290 Infinity LC (Agilent, Santa Clara, CA, USA). In a typical run, 4 μL of the blood serum sample was directly loaded without further treatment onto a Zorbax column (C18, 100 mm × 2.1 mm, 1.8 μm, Agilent, Santa Clara, CA, USA). The separation was achieved under the column temperature of 40 °C using a controlled gradient of mobile phase A consisting of 0.1% (v/v) formic acid in water and mobile phase B, consisting of 0.1% formic acid in acetonitrile at a flow rate of 0.4 mL/min. The gradient flow was first set at 5% (v/v) B for 2 min, linearly increased to 95% B over 11 min, and maintained at this composition for an additional 2 min.

MS signals in the m/z range of 50–1000 were separately acquired under positive-ion and negative-ion mode using nitrogen as both the nebulizing gas and cone gas. The instrument settings were as follows. For positive-ion mode: source temperature – 100 °C; capillary voltage – 4 kV; cone voltage – 35 kV; extraction voltage – 4 V; desolvation temperature – 350 °C; cone gas flow – 50 L/h; desolvation gas flow – 600 L/h; scan time – 0.03 s; inter-scan delay – 0.02 s. For negative-ion mode, the capillary voltage, cone voltage, desolvation temperature and desolvation gas flow were set to 3.5 kV, 50 kV, 350 °C and 700 L/h, respectively, while other instrument parameters were unchanged. For lock mass correction, a standard leucine-enkephalin sample was used, which should generate an [M+H]^+^ ion with a theoretical m/z value of 556.2771 Da under positive-ion mode and an [M-H]^−^ ion of 554.2615 Da under negative-ion mode. Data reproducibility was verified by performing the same LC-MS experiment on the abovementioned quality control sample and subsequently conducting coefficient of variation (CV) analysis.

### Statistical analysis

The raw LC-MS data sets obtained under the positive- and negative-ion modes were first subjected to unit variance scaling and mean-centered using XCMSonline (https://xcmsonline.scripps.edu/). All samples were normalized based on the total area and then imported into SIMCA-p 12.0 Software (Umetrics AB, Umea, Sweden) for multivariate statistical analysis. The area of peaks that failed to be detected was transferred to 0.01. In total, three analytical methods, including principle component analysis (PCA), partial least squares discriminant analysis (PLS-DA) and orthogonal partial least squares discriminant analysis (OPLS-DA), were used to identify the global metabolic differences between the patient group and the control group. The variable importance for the projection (VIP) value for each identified metabolite came from the validated OPLS-DA model^10^. Meanwhile, Student’s T-test was performed on each metabolite using SPSS 22.0 software (SPSS, Inc., Chicago, IL, USA) to calculate the p-value. The screening criteria of VIP > 1 and p < 0.05 were applied to the final selection of a panel of metabolites for further analysis of their potential implications in the pathogenesis of BAV. Cross-validation of PLS–DA was performed by employing R software.

### Chemical identification of the selected metabolites

Data matching was performed by querying the high performance liquid chromatography-mass spectrometry (HPLC-MS) results of each selected metabolite against the METLIN database (http://metlin.scripps.edu/) to determine its chemical identity. The list of selected metabolites was imported into MetaboAnalyst 3.0 (http://www.metaboanalyst.ca/) for a variety of functional enrichment analysis.

### Model construction and validation for the prediction of BAV

Correlation analysis was conducted by calculating the Spearman correlation coefficient. Binary logistic regression analyses were employed to construct the models for BAV diagnosis and prediction. Probability values were yielded from the diagnostic models, which were subsequently utilized as new input variables for the receiver operating characteristic (ROC) curve analysis. Model accuracy assessment was conducted by constructing ROC curves. Differences among the areas under curve (AUCs) were evaluated by z-test. The probability cut-off thresholds for the optimal combination of sensitivity and specificity for all generated models were calculated by the Youden index. All *P-* values were 2-sided, and values < 0.05 were considered statistically significant.

The diagnostic models were validated by the standard diagnostic analysis of sensitivity, specificity, as well as positive and negative predictive values. And the performers were blind to the clinical information.

## Results

### Overall description of the study population

A total of 76 patients and 40 healthy individuals were initially enrolled in this study. Seven patients were excluded due to medicine usage and another nine based on exclusion criteria. The final study cohort, comprising a total of 100 subjects, was equally divided between an estimation group and a validation group, each consisting of 30 patients and 20 healthy participants as controls. Table 1 summarized the demographic and echocardiographic data for all participants, as well as the prevalence of aortic valve dysfunction, aortopathy and other concomitant abnormalities such as atrial fibrillation and atrioventricular block in different groups.

### The metabolic profiles of BAV patients versus healthy controls

An untargeted metabolomics approach based on liquid chromatography coupled to electrospray ionization quadrupole time-of-flight MS was employed to analyze the relative abundance of metabolites in both the BAV patients and healthy participants. The MS-based platform in theory can detect more than 5000 metabolite features, defined as molecules each with a unique set of mass/charge ratio and retention time. The relative abundances of all identified metabolites were determined based on their respective peak areas and then used to construct global metabolic profiles for all subjects. An overall peak detection of the metabolic profiles in members of the patient group and those of the control group led to the identification of 738 features under positive-ion mode and 1475 features under negative-ion mode (See Supplement Fig. 1). The analysis of the quality control sample found an average CV of 11.5% for the integrated peak area, confirming the excellent data reproducibility and robustness of the experimental method.

### Identification of putative BAV metabolite biomarkers

**Figure 2:**
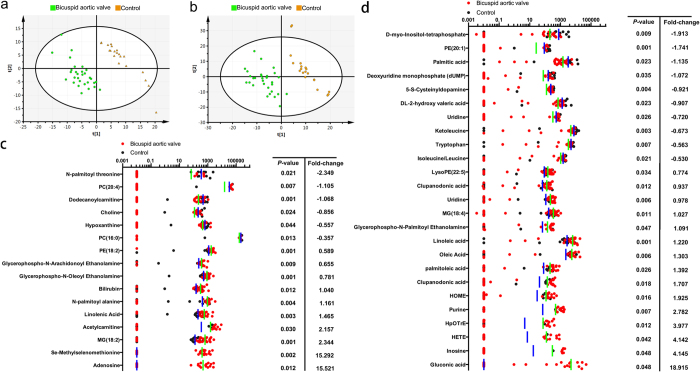
The metabolic profiles of BAV patients exhibited a distinct pattern characterized by changes in the levels of certain serum metabolites. The data points representing the patient group were shown to be clustered together and separated from those of the control group in both the PLS-DA score plots constructed for positive- (**a**) and negative-ion (**b**) mode. The 16 metabolites under positive-ion mode (**c**) and 25 under negative-ion mode (**d**) that met the selection criteria of VIP > 1 and p < 0.05 were quantified in all subjects, and their levels, defined as the median values of the normalized peak intensities yielded by the LC-MS measurements, were represented by the data points and bars (green for patients and blue for healthy subjects), and plotted on a logarithmic scale. The fold-change value of each metabolite shown denoted the difference between the averaged normalized peak intensity for the patient group over that for the control group. Identification was based on accurate mass and MS/MS data. BAV = bicuspid aortic valve; PLS-DA = partial least squares discriminant analysis; VIP = importance in the projection; LC-MS = liquid chromatography-mass spectrometry; MS/MS = tandem mass spectrometry.

The normalized integration values of all metabolite features were subjected to unit variance scaling and mean centering before being evaluated through Principle Component Analysis (PCA) in Simca-P to ascertain whether there were statistically significant differences between the metabolic profiles of the patient group and those of the control group. QC samples were used to confirm the reliability of the analytical method (For representative base peak chromatograms of the QC samples for positive- and negative-ion modes, see Supplement Fig. 2). As evidenced by the PCA plots (See Supplement Fig. 3), the QC samples were tightly clustered together and the two study groups were separated from each other, suggesting a possible association between BAV and certain metabolic perturbations. Furthermore, 91% of all detected metabolites showed a CV below 20%, indicating negligible random errors and satisfactory data reproducibility. The PLS-DA score plots also indicated a clear separation between the BAV patients and controls (Fig. 2a,b). The Q2 values of the PLS-DA models were 45.7% for positive-ion mode and 52.5% for negative-ion mode, both of which exceeded the 40% threshold indicative of model validity (See Supplement Fig. 4). To identify and select diagnostically important metabolic features, the VIP and *P* values of each metabolite were determined from OPLS-DA and t-test, respectively. In the end, 16 metabolite features (2.17%) under positive-ion mode and 25 (1.76%) under negative-ion mode met the selection criteria of VIP > 1 and *P*-value < 0.05, which denoted that their levels in the BAV patients were significantly different from those in the healthy participants (Fig. 2c,d). These metabolite features were thus selected to be further analyzed for their possible roles in the pathogenesis of BAV.

### Abnormal metabolism in bicuspid aortic valve

The clinical implications of the 41 metabolites meeting the selection criteria were investigated by MetaboAnalyst 3.0, which calculated the enrichment value and identified the most relevant metabolic roles for each query compound (Table 2). The pathways related to purine metabolism and fatty acid biosynthesis were directly identified as those that might have been perturbed by the development of BAV. Consistent with these results, the analysis also pointed to signs of redox imbalance and deficiency in energy production, which could be rationalized by the fact that purine is a key component of ATP, and fatty acids are the primary energy source for myocardial cells. In addition, two single nucleotide polymorphisms (SNP), rs10827283 and rs1562861, were suggested to have the highest correlation with the observed metabolic perturbations in BAV patients. Both SNPs are characterized by a single A-to-G mutation, which was consistent with the genetic consequence of the predicted nicotinamide adenine dinucleotide phosphate (NADPH)-dependent guanosine 5′-monophosphate (GMP) reductase abnormality.

### Identification of the best BAV diagnostic indicators and construction of a diagnostic model based on logistical regression analysis

Based on correlation analysis (See Supplement Table 1), three metabolites, glycerophospho-N-oleoyl ethanolamine (GNOE), monoglyceride (MG) (18:2) and phosphatidylethanolamine (PE) (18:2), were shown to have the best correlation with BAV (r = 0.4711 (GNOE), 0.4637 (MG, 18:2), 0.4537 (PE, 18:2)), making them the most accurate predictive risk factors of the disease. In addition, logistical regression analysis was used to generate a BAV diagnostic model that incorporated only the levels of GNOE and MG (18:2), with the following expression:


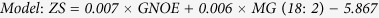


### Model accuracy assessment of GNOE, MG (18:2), PE (18:2) and ZS using the estimation group

**Figure 3:**
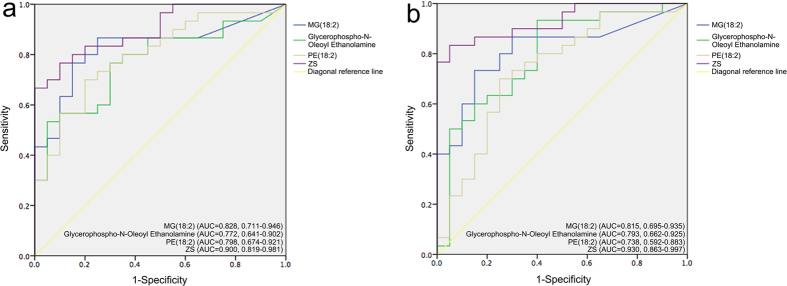
ROC of MG (18:2), Glycerophospho-N-Oleoyl Ethanolamine, PE (18:2) and ZS in the prediction of patients with bicuspid aortic valve in estimation (**a**) and validation (**b**) groups. AUC = area under curve; ZS = Zhongshan; ROC = receiver operating characteristic curves.

The predictive capacities of GNOE, MG (18:2), PE (18:2) and ZS model were assessed by ROC curve analysis and compared to each other. Based on the constructed ROC curves, the AUC values of MG (18:2), GNOE, PE (18:2) and ZS were calculated to be 0.828, 0.772, 0.798 and 0.900, respectively, for BAV prediction (Fig. 3a). The AUC values of MG (18:2), GNOE, PE (18:2) and ZS in the validation group were 0.815, 0.793, 0.738 and 0.930, respectively, which were all similar to their counterparts in the estimation group (Fig. 3b). Therefore, ZS model, with its highest AUC value, was the best performer in the prediction of BAV for the estimation group. Although ZS showed no statistical differences compared to the three metabolite candidates (*p > *0.05), it exhibited an obvious higher AUC in the prediction of BAV.

### Comparison of GNOE, MG (18:2), PE (18:2) and ZS in BAV prediction for the validation group

The predictive capacities of GNOE, MG (18:2), PE (18:2) and ZS were further evaluated by the use of a validation group that also consisted of 30 BAV patients and 20 healthy adults. The sensitivities, specificities, as well as negative and positive predictive values for all four models were calculated based on their respective cut-offs, as summarized in Table 3. Among the 20 healthy adults, 18 (90%) had ZS scores below the optimal cutoff of −0.1634, 15 (75%) had PE (18:2) scores below the cutoff of 1447.81, 14 (70%) had GNOE scores lower than the cutoff of 227.56, and 17 (85%) had MG (18:2) scores less than the cutoff of 237.9. On the other hand, 23 out of the 30 (76.7%) BAV patients had ZS scores above the cutoff, followed by GNOE (23, 76.7%), MG (18:2) (23, 76.7%), and PE (18:2) (22, 73.3%). In both cases, ZS model outperformed all three candidate metabolites in prediction accuracy by demonstrating the highest AUC value and the lowest likelihood of misdiagnosing healthy participants as BAV patients. In comparison, although GNOE seemed to have predicted the correct number of patients, this was in fact partly attributable to its propensity to misidentify BAV patients as healthy adults, and vice versa.

## Discussion

To our knowledge, this study constitutes the first report on the application of metabolomics to the study of BAV and its underlying pathogenesis. From a pool of 738 metabolites detected under positive-ion mode and 1475 metabolites under negative-ion mode by LC-MS, we identified a total of 41 features whose average levels exhibited statistically significant differences between the patient group and the control group. Subsequent correlation analysis revealed that GNOE, MG (18:2) and PE (18:2) had the highest correlation with BAV. A predictive model (ZS) was then developed based on the concentrations of GNOE and MG (18:2), and achieved greater accuracy than the three individual metabolites mentioned above in the diagnosis of BAV for the validation group.

Metabolomics profiling has recently emerged as a powerful screening method that can aid in the prompt diagnosis of various heart diseases. The application of metabolomics analysis to a cohort of 39 chronic heart failure (CHF) patients and 15 healthy individuals found 18 metabolites that showed different expression patterns between the two groups, out of 22 reliably detected peaks. The constructed OPLS-DA model was shown to be able to predict CHF with 92.31% sensitivity and 86.67% specificity^11^. In another study, a combination of ultra-performance liquid chromatography (UPLC) and MS was employed in search for novel metabolite biomarkers with predictive value for the diagnosis of ST-segment elevation myocardial infraction in young patients. In total, 24 different metabolites were identified out of approximately 400 statistically confirmed candidates, among which ceramides [Cer(d18:0/16:0), Cer(t18:0/12:0)] and sphinganine achieved 75–100% sensitivity and 42.11–78.95% specificity in predicting major adverse cardiovascular events in young patients that had been discharged^12^. Compared to these studies, our analytic method detected significantly more metabolic signatures, leading to the identification of an increased number of differentiating metabolites. In addition, our ZS model showed comparable predictive power, reflected by its high sensitivity and specificity scores. The statistical validity of our predictive models was also supported by the satisfactory Q2 values and the similar AUC scores between the estimation and validation groups.

The first observation from a quick overview of the 41 identified metabolites was altered levels of endocannabinoids and other cannabimimetic compounds in the BAV patients. Particularly, two of the three candidate metabolites that we identified as having the best predicting power, PE (18:2) and glycerophospho-N-ethanolamine, belonged to this category. Similar patterns were also discovered under negative-ion mode. It is widely agreed that endocannabinoids and cannabimimetic compounds can participate in stress response pathways to counter various forms of cardiovascular dysfunction, including myocardial infarction (MI) and circulatory shock, by promoting the relaxation of coronary and other arteries^13^. Of particular relevance to this study, a variety of N-acylethanolamines have been confirmed to be peroxisome proliferator-activated receptor (PPAR) ligands, which play key roles in essential physiological processes such as lipid metabolism and inflammation response^14^,^15^. It has been reported that N-oleoyl ethanolamine could activate PPAR-alpha receptor and promote the release of glycerol and fatty acids from rat adipocytes^16^. The results of these studies are perfectly consistent not only with the fact that glycerophospho-N-oleoyl ethanolamine was revealed in this study to be one of the best predicting markers for BAV, but also with the detection of elevated levels of certain types of free fatty acids and monoglycerides in the patients.

Fatty acids are the primary energy source for the heart and have been associated in many studies with cardiovascular diseases^17^,^18^. Perturbation of fatty acid synthesis and metabolism could lead to abnormal levels of free carboxylates. This was supported by the finding that the BAV patients and healthy participants of this study had significantly different levels of acetylcarnitine and dodecanonylcarnitine. Carnitine and its derivatives are well-known for their role in the transport of fatty acids to mitochondria for beta-oxidation^19^. As a result, they have also been explored as potential biomarkers for predicting cardiovascular anomalies^20^. In fact, altered levels of circulating carnitine and its acyl derivatives have been observed in HF and MI patients^21^,^22^. In particular, a recent metabolomics study reported a prognostic model that included butyrylcarnitine as an essential component, which achieved better results in predicting the outcome of patients with HF compared to conventional biomarkers^22^.

Additional signs of oxidative stress in the BAV patients were reflected by the changes in the levels of purine-based metabolites, which were suggested as diagnostic indicators for various cardiovascular disorders in a number of studies. Kugler G. examined a cohort of patients with coronary artery diseases and found increased levels of inosine and hypoxanthine in their coronary venous blood^23^. In another study, Harmsen E. *et al*. observed significantly increased levels of hypoxanthine, but not of adenosine, inosine and xanthine, in the blood samples collected from ischemic heart patients^24^. In agreement with these findings, our metabolomics results revealed substantially higher levels of adenosine, inosine and purine, as well as a moderately lower concentration of hypoxanthine in the BAV patients of this study, which were signs of ATP degradation that possibly arose from a lack of oxygen supply.

The main limitation of the current study lies in the relative small sample size. Consequently, further investigations involving larger populations are necessary in order for our results and model to be generally applicable to the broader community. In addition, the specificity and sensitivity values of the ZS model are slightly lower than those of conventional echocardiogram- and CT imaging-based tests. Further metabolic profiling studies are also necessary to ascertain whether our models can differentiate between BAV and other heart diseases that share similar underlying metabolic disturbances. For example, since dysregulation of fatty acid metabolism is a common theme in MI and HF, a comparative study on these pathologies and BAV would be needed to determine their respective metabolomics signatures. Nevertheless, our serum-based diagnostic method is particularly suitable for use in community clinics and other medical institutes with inadequate equipment and funding, thanks to the easy availability of blood samples and the relatively low capital requirement for the analysis of small-molecule metabolites. Furthermore, the rapid advances in the area of high-throughput assays and robot-assisted automation protocols would allow the routine screening of large populations, which in turn would greatly facilitate the epidemiological study of BAV and the understanding of its underlying mechanisms. Efforts to enrollment of more participants for further assessment of ZS model and the mechanisms behind the relationship between BAV and significantly different metabolites are currently underway in our laboratory.

## Additional Information

**How to cite this article**: Wang, W. *et al*. Analysis of Serum Metabolites to Diagnose Bicuspid Aortic Valve. *Sci. Rep.* **6**, 37023; doi: 10.1038/srep37023 (2016).

**Publisher’s note**: Springer Nature remains neutral with regard to jurisdictional claims in published maps and institutional affiliations.

## Supplementary Material

Supplementary Information

## Figures and Tables

**Table 1:** Baseline characteristics of the subjects in the estimation and validation groups.

**Variables**	**Estimation group**			**Validation group**		
	**Bicuspid aortic valve**	**Control**	***P*****-value**	**Bicuspid aortic valve**	**Control**	***P*****-value**
Age (years)	51.7 ± 16.7	53.9 ± 12.1	0.6149	49.7 ± 10.5	51.3 ± 12.7	0.6279
male gender	20 (66%)	13 (65%)	0.570	19 (63%)	13 (65%)	0.574
Abnormal valve function	27 (90%)	0 (0%)	<0.0001	28 (93%)	0 (0%)	<0.0001
Concomitant aortopathy	20 (66%)	0 (0%)	<0.0001	22 (73%)	0 (0%)	<0.0001
Concomitant other cardiovascular structural defects	0 (0%)	0 (0%)	1.00	0 (0%)	0 (0%)	1.00
Atrial fibrillation	0 (0%)	0 (0%)	1.00	0 (0%)	0 (0%)	1.00
Atrioventricular block	0 (0%)	0 (0%)	1.00	0 (0%)	0 (0%)	1.00
BMI	21.87 ± 1.41	21.13 ± 1.67	0.0978	20.91 ± 1.75	21.23 ± 1.32	0.4901
Ejection fraction (%)	62.8 ± 9.55	62.9 ± 7.83	0.969	59.1 ± 10.69	61.7 ± 9.73	0.8478
Hypertension	9 (30%)	7 (25%)	0.763	10 (33%)	7 (35%)	0.9025
Smoking	11 (37%)	8 (40%)	1.0	9 (30%)	7 (35%)	0.7103
Surgical repair	30 (100%)	0 (0%)	<0.0001	30 (100%)	0 (0%)	<0.0001

Continuous variables are expressed as means and standard deviation. Aortic regurgitation and/or aortic stenosis with at least moderate severity. Aortic dilation ≥40 mm, affecting any part of the aorta from sinus of valsalva to proximal descending aorta.

**Table 2:** Enrichment and pathway analysis.

**Category**		**Total**	**Hits**	***P*****-value**
**Disease-associated**				
	critical illness (cardiogenic shock)	6	3	0.0016
	Lesch-Nyhan syndrome	5	2	0.0189
	metabolites affected by exercise	5	2	0.0189
	early markers of myocardial injury	14	3	0.0227
	Crigler-Najjar syndrome | glucose-6-phosphate dehydrogenase deficiency | intoxication acetaminophen [dd] | pyruvate kinase deficiency	1	1	0.0464
**Enzyme-associated**				
	GMP reductase	6	3	0.0101
	guanine phosphoribosyltransferase	6	3	0.0101
	carnitine O-palmitoyltransferase	3	2	0.0212
	Beta oxidation of fatty acid	3	2	0.0212
	transport into the mitochondria (carnitine)	4	2	0.0400
	methenyltetrahydrofolate cyclohydrolase	4	2	0.0400
	carnitine transferase	4	2	0.0400
**SNP-associated**				
	rs10827283 | rs9663087	5	2	0.0385
	rs1562861	5	2	0.0385
	rs2039334	5	2	0.0385
**Location-associated**				
	skeletal muscle	45	4	0.0433
**Pathway**				
	Fatty acid biosynthesis	49	3	0.0052
	Purine metabolism	92	3	0.0290

**Table 3:** Model validation and comparison with regard to identifying BAV patients in the validation group.

**Metabolomic target**	**Cutoffs**	**Patients classified N (%)**	**Diagnostic accuracy**		**AUC**
MG (18:2)	237.9	28 (56)	Sen (%)	76.7	0.815
			Spe (%)	85.0	
			PPV (%)	80.0	
			NPV (%)	80.0	
Glycerophospho-N-Oleoyl Ethanolamine	277.56	30 (60)	Sen (%)	76.7	0.793
			Spe (%)	70.0	
			PPV (%)	76.7	
			NPV (%)	65.0	
PE (18:2)	1447.81	35 (70)	Sen (%)	73.3	0.738
			Spe (%)	75.0	
			PPV (%)	83.3	
			NPV (%)	50.0	
ZS	−0.1634	27 (54)	Sen (%)	76.7	0.93
			Spe (%)	90.0	
			PPV (%)	80.0	
			NPV (%)	85.0	

Sen = sensitivity; Spe = specificity; PPV = positive predictive value; NPV = negative predictive value.
