# Supplementary material for: Analysis of Serum Metabolites to Diagnose Bicuspid Aortic Valve
Source: Sci Rep. 2016 Nov 15;6:37023. doi: 10.1038/srep37023 (PMC5109472; doi:10.1038/srep37023)
Supplement: Supplementary Information [file srep37023-s1.pdf]

# Analysis of Serum metabolites to Diagnose Bicuspid Aortic Valve

Wenshuo Wang[1, ‡], Aikebaier Maimaiti[1, ‡], Yun Zhao[1], Lingfei Zhang[2], Hongyue Tao[3], Hui Nian[1], Limin Xia[1], Biao Kong[4], Chunsheng Wang[1, *], Mofang Liu[2, *], Lai Wei[1, *]

1. Department of Cardiac Surgery, Zhongshan Hospital, Fudan University. 200032, Shanghai, China

2. State Key Laboratory of Molecular Biology, Shanghai Key Laboratory of Molecular Andrology, Institute of Biochemistry and Cell Biology, Shanghai Institutes for Biological Sciences, Chinese Academy of Sciences, 200031, Shanghai, China.

3. Department of Radiology, Huashan Hospital, Fudan University. 200040, Shanghai, China

4. Department of Chemistry, Laboratory of Advanced Materials, Fudan University, 200433, Shanghai, China

‡ These authors contributed equally to this work

* Corresponding authors:

Lai Wei

Telephone Number: +86 (21) 64041990
Fax Number: +86 (21) 64041990
Email address: weilai_zs@sina.com
Mofang Liu
Telephone Number: +86 (21) 54921146
Fax Number: +86 (21) 54921146
Email address: liumofang@sohu.com
Chunsheng Wang
Telephone Number: +86 (21) 64041990
Fax Number: +86 (21) 64041990
Email address: zswangchunsheng@sina.com

**Supplementary Material**

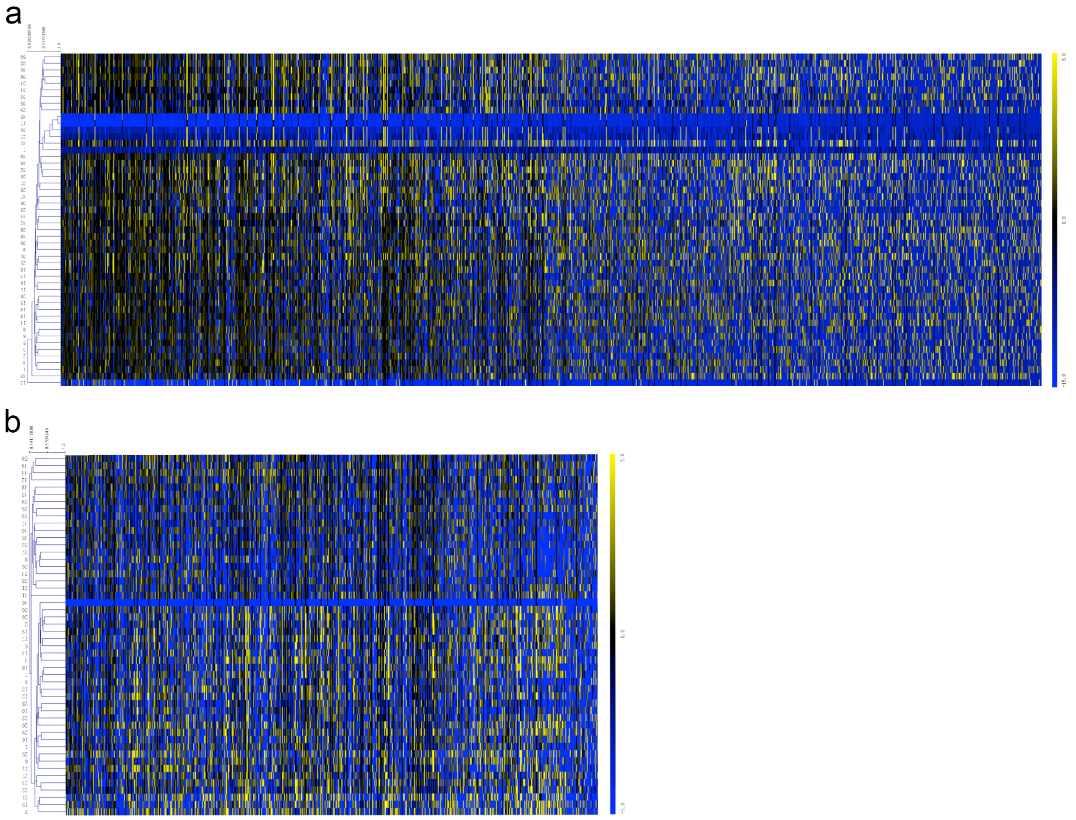

**Figure 1.** The metabolic profiles of BAV patients versus healthy participants. Heat maps of metabolite features identified from members of the patient group and control group under negative-ion mode (a) and positive-ion mode (b) were shown. The data were displayed on a log2 scale and clustered based on Spearman distance.

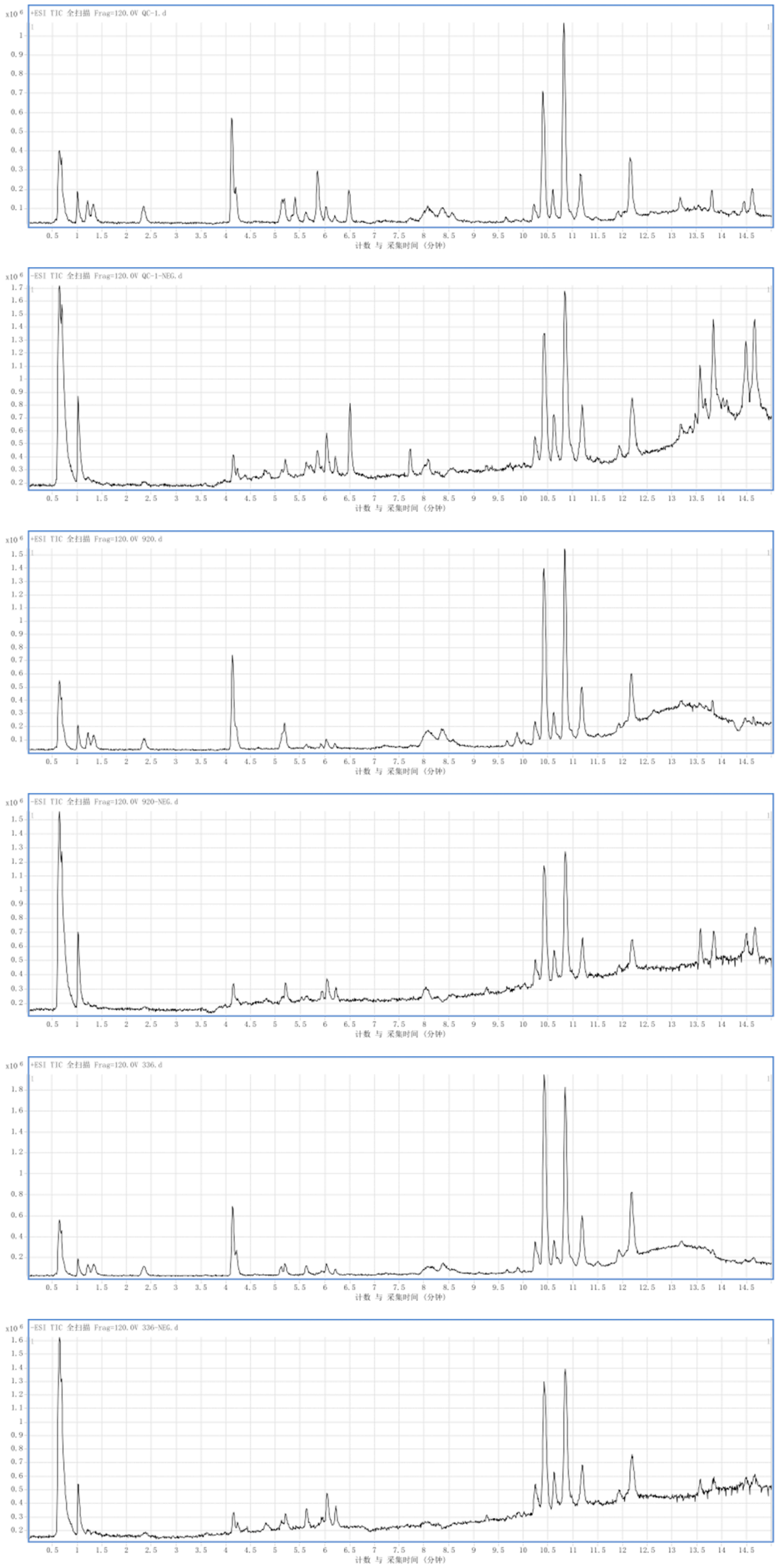

Total Ions Chromatograph of QC in positive mode

Total Ions Chromatograph of QC in negative mode

Total Ions Chromatograph of patients with BAV in positive mode

Total Ions Chromatograph of patients with BAV in negative mode

Total Ions Chromatograph of control in positive mode

Total Ions Chromatograph of control in negative mode

**Figure 2.** The TIC of all groups in positive- and negative-ion modes. TIC = total ions chromatography

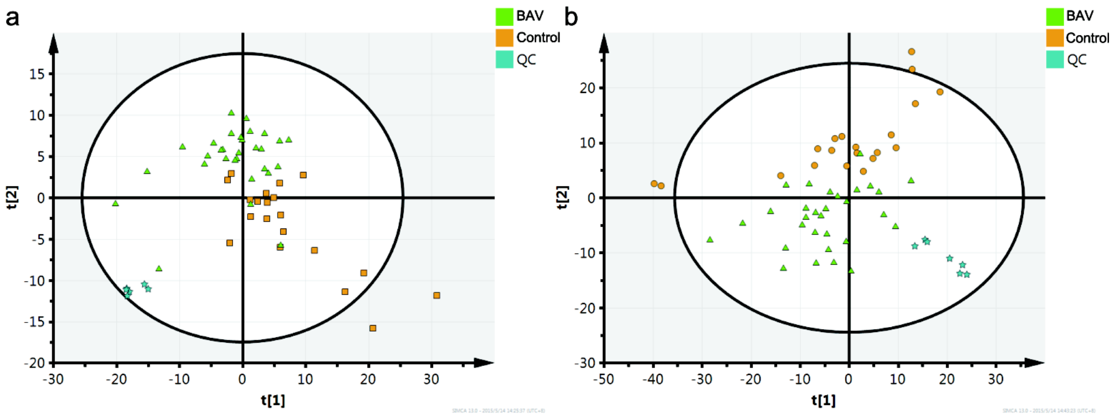


**Figure 3.** The PCA scores of patients with BAV and controls in positive- (a) and negative-ion (b) modes. PCA = Principle Component Analysis. BAV = bicuspid aortic valve. QC = Quality Control.

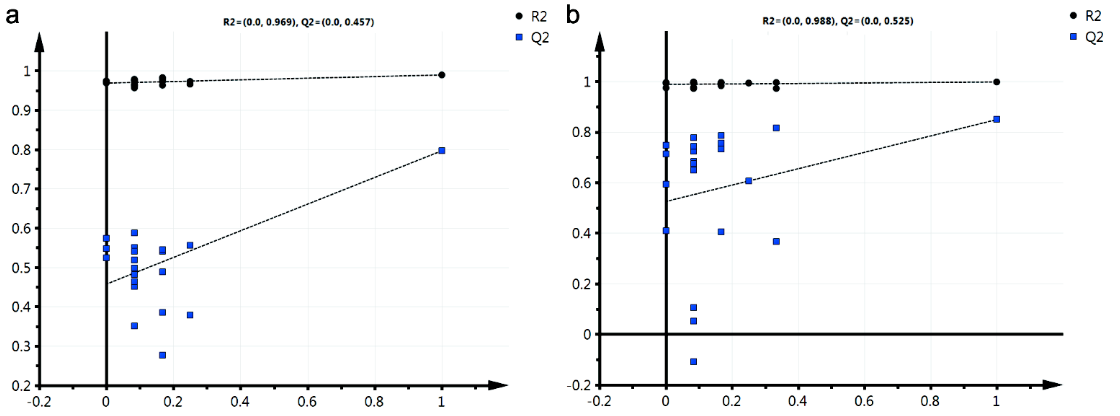


**Figure 4.** The validation of PLS-DA models in positive (a) and negative (b) mode. PLS-DA = Partial Least Squares Discriminant Analysis.

| Metabolites | R | *P*-value |
|---|---|---|
| Gluconic acid | 0.2813 | 0.0478 |
| Inosine | 0.2805 | 0.0485 |
| HETE | 0.2884 | 0.0422 |
| HpOTrE | 0.3542 | 0.0116 |
| Purine | 0.3762 | 0.0071 |
| HOME | 0.3379 | 0.0164 |
| Clupanodonic acid | 0.3331 | 0.0181 |
| palmitoleic acid | 0.3154 | 0.0257 |
| Oleic Acid | 0.3855 | 0.0057 |
| Linoleic acid | 0.4484 | 0.0006 |
| Glycerophospho-N-Palmitoyl Ethanolamine | 0.2828 | 0.0466 |
| MG(18:4) | 0.3566 | 0.0110 |
| Uridine | 0.3811 | 0.0063 |
| Clupanodonic acid | 0.3513 | 0.0124 |
| LysoPE(22:5) | 0.3000 | 0.0343 |
| Isoleucine/Leucine | -0.3263 | 0.0207 |
| Tryptophan | -0.3797 | 0.0065 |
| Ketoleucine | -0.4126 | 0.0029 |
| Uridine | -0.3152 | 0.0258 |
| DL-2-hydroxy valeric acid | -0.3203 | 0.0233 |
| 5-S-Cysteinyldopamine | -0.4024 | 0.0038 |
| Deoxyuridine monophosphate (dUMP) | -0.2984 | 0.0353 |
| Palmitic acid | -0.3217 | 0.0227 |
| PE(20:1) | -0.4385 | 0.0014 |
| D-myo-Inositol-tetraphosphate | -0.3656 | 0.0090 |
| Adenosine | 0.3522 | 0.0121 |
| Se-Methylselenomethionine | 0.4308 | 0.0018 |
| MG(18:2) | 0.4637 | 0.0007 |
| Acetylcarnitine | 0.3079 | 0.0296 |
| Linolenic Acid | 0.4093 | 0.0032 |
| N-palmitoyl alanine | 0.4042 | 0.0036 |
| Bilirubin | 0.3513 | 0.0124 |
| Glycerophospho-N-Oleoyl Ethanolamine | 0.4711 | 0.0006 |
| Glycerophospho-N-Arachidonoyl Ethanolamine | 0.3668 | 0.0088 |
| PE(18:2) | 0.4537 | 0.0009 |
| PC(16:0) | -0.3496 | 0.0128 |
| Hypoxanthine | -0.2861 | 0.0440 |
| Choline | -0.3183 | 0.0243 |
| Dodecanoylcarnitine | -0.4446 | 0.0012 |
| PC(20:4) | -0.3795 | 0.0066 |
| N-palmitoyl threonine | -0.3251 | 0.0212 |

**Table 1.** The correlation of metabolites with BAV. BAV = bicuspid aortic valve
